# Presence of autism, hyperserotonemia, and severe expressive language impairment in Williams-Beuren syndrome

**DOI:** 10.1186/2040-2392-4-29

**Published:** 2013-08-23

**Authors:** Sylvie Tordjman, George M Anderson, David Cohen, Solenn Kermarrec, Michèle Carlier, Yvan Touitou, Pascale Saugier-Veber, Céline Lagneaux, Claire Chevreuil, Alain Verloes

**Affiliations:** 1 Department of Child and Adolescent Psychiatry, Centre Hospitalier Guillaume Regnier and Medical School of the University of Rennes 1, Rennes 35000, France; 2 Laboratoire de la Psychologie de la Perception, CNRS UMR 8158, Centre Biomédical des Saints Pères, 75006 Paris, France; 3 The Child Study Center, Yale University School of Medicine, New Haven CT 06511, USA; 4 Université Pierre et Marie Curie and CNRS UMR 7222, Paris, France; 5 Laboratoire de Psychologie Cognitive, Aix-Marseille University, CNRS UMR 7290, Marseille, France; 6 Chronobiology Unit, Rothschild Foundation, Paris, France; 7 Department of Genetics, Rouen University Hospital, Rouen, France; 8 Department of Genetics, AP-HP-Robert Debré University Hospital, Paris, France; 9 INSERM U676, AP-HP-Robert Debré University Hospital, Paris, France

**Keywords:** 7q11.23, Autistic disorder, Serotonin, Gene–environment interactions, Gene–phenotype correlations, Genetic background

## Abstract

**Background:**

Deletion of the Williams-Beuren syndrome (WBS) critical region (WBSCR), at 7q11.23, causes a developmental disorder commonly characterized by hypersociability and excessive talkativeness and often considered the opposite behavioral phenotype to autism. Duplication of the WBSCR leads to severe delay in expressive language. Gene–dosage effects on language development at 7q11.23 have been hypothesized.

**Methods:**

Molecular characterization of the WBSCR was performed by fluorescence *in situ* hybridization and high-resolution single-nucleotide polymorphism array in two individuals with severe autism enrolled in a genetic study of autism who showed typical WBS facial dysmorphism on systematic clinical genetic examination. The serotonin transporter promoter polymorphism (*5-HTTLPR*, locus *SLC6A4*) was genotyped. Platelet serotonin levels and urinary 6-sulfatoxymelatonin excretion were measured. Behavioral and cognitive phenotypes were examined.

**Results:**

The two patients had common WBSCR deletions between proximal and medial low copy repeat clusters, met diagnostic criteria for autism and displayed severe impairment in communication, including a total absence of expressive speech. Both patients carried the *5-HTTLPR ss* genotype and exhibited platelet hyperserotonemia and low melatonin production.

**Conclusions:**

Our observations indicate that behaviors and neurochemical phenotypes typically associated with autism can occur in patients with common WBSCR deletions. The results raise intriguing questions about phenotypic heterogeneity in WBS and regarding genetic and/or environmental factors interacting with specific genes at 7q11.23 sensitive to dosage alterations that can influence the development of social communication skills. Thus, the influence of WBSCR genes on social communication expression might be dramatically modified by other genes, such as *5-HTTLPR*, known to influence the severity of social communication impairments in autism, or by environmental factors, such as hyperserotonemia, given that hyperserotonemia is found in WBS associated with autism but not in WBS without autism. In this regard, WBS provides a potentially fruitful model with which to develop integrated genetic, cognitive, behavioral and neurochemical approaches to study genotype–phenotype correlations, possible gene–environment interactions and genetic background effects. The results underscore the importance of considering careful clinical and molecular genetic examination of individuals diagnosed with autism.

## Background

Williams-Beuren syndrome (WBS) is a developmental disorder caused by a hemizygous recurrent deletion of the WBS critical region (WBSCR) at chromosomal band 7q11.23, which includes *ELN* (gene coding for elastin) and 27 additional coding genes [1]. The common deletion results from recombination between misaligned low copy repeat (LCR) sequences flanking the critical region. Three LCR clusters have been delineated: centromeric, medial and telomeric. Each LCR cluster is broken down in highly homologous subregions (blocks A, B and C). The deletions arise as a consequence of a nonallelic homologous recombination. The deletion occurs between proximal and medial LCRs in about 95% of WBS cases, and it occurs between proximal and distal LCRs in the remaining cases. The size of the deletion is approximately 1.55 Mb for the small deletion and approximately 1.84 Mb for the larger one [1-5]. However, the precise size depends upon the exact position of the breakpoints in each block.

The physical WBS phenotype includes typical facial dysmorphism (elfin-like face), vascular stenoses (most commonly aortic stenosis), infantile hypercalcemia, dental problems, kidney abnormalities, feeding and sleep disturbances, abnormal gait and developmental delay with short stature [6,7]. The characteristic cognitive and behavioral profile includes some strengths in socialization (overfriendliness and enhanced social interest) and communication (excessive talkativeness and hyperverbal speech) with relatively good short-term verbal memory, contrasting with a common mild to moderate intellectual disability (it is noteworthy that some patients with WBS have been reported to show severe intellectual disability [8]) and severe impairment in visuospatial abilities associated with hyperacusis, peer interaction difficulties, general anxiety and behavioral problems such as hyperactivity [9-13].

The roles played by most genes deleted in the WBSCR are largely unknown. However, deletion of the elastin gene (*ELN*) is clearly involved in the vascular anomalies [14], and the *LIMK1*, *GTF2I* and *GTFIIRD1* genes have been implicated in cognitive developmental delay and visuospatial deficiency [3,7,15]. Recently, duplication of the WBSCR has been reported to be associated with intellectual disability, severe delay in expressive language and autism spectrum disorders, suggesting that specific genes within WBSCR can influence language and social development through gene–dosage effects [16-19].

Here we report on two unrelated Caucasian individuals with typical WBSCR deletion and severe autistic disorder, including a total absence of expressive language, found during a systematic clinical genetic examination of a cohort of 71 individuals with autistic disorder enrolled for a genetic study of autism. The existence of such individuals raises questions about the common idea that WBS (regarding its characteristics of overfriendliness and excessive talkativeness) represents the opposite phenotype of autism (a developmental disorder characterized by social and communication deficits and stereotypies) and suggests that genetic background or epistatic effects can markedly influence the effects of WBSCR deletion on the development of social communication skills. To study further the characteristics of these two individuals, behavioral and cognitive phenotypes were examined by assessing severity of impairments in the main behavioral domains of autistic disorder and in cognitive functioning. Genetic analyses were performed by the molecular characterization of the WBSCR and by genotyping the serotonin transporter promoter polymorphism (*5-HTTLPR* at 17q11.2, locus *SLC6A4*). Given the well-replicated hyperserotonemia of autism [20,21] and multiple reports [22,23] of decreased melatonin production in autism (melatonin is a pineal neurohormone directly synthesized from serotonin), neurochemical phenotype was assessed by measuring whole-blood serotonin and urinary excretion of the melatonin metabolite 6-sulfatoxymelatonin. As these two neurochemical measures are the most well-established autism biomarkers (albeit without sufficient sensitivity or specificity to be used alone in assessing autism risk), their measurement was included in the characterization of the WBS individuals under study.

## Methods

### Patients and clinical genetic examination

On the basis of direct clinical observation of the two patients by two independent child psychiatrists, the diagnosis of autistic disorder was made according to the criteria set forth in the *Diagnostic Manual of Mental Disorders, Fourth Edition–Text Revision* (DSM-IV-TR) [24], and the International Classification of Diseases, 10th Revision (ICD-10) [25]. The protocol was approved by the ethics committee of Bicêtre Hospital (CPP of Bicêtre). Written informed consent was obtained from the participants’ parents and guardians, including a written consent to publish. The clinical characteristics and physical features of both patients are presented in Table 1.

**Table 1:** **Demographic information, physical features and medical information**^**a**^

**Characteristics**	**Patient 1**	**Patient 2**
Demographics		
Sex	M	M
Age (yr; mo)	17;6	19;2
Age autistic disorder reported by a professional (yr; mo)	0;11	2;11
Age at referral to an autism center (yr; mo)	3;6	5;9
Age at WBS diagnosis (yr; mo)	17;6	19;2
Clinical genetic examination and physical features		
Birth height	45 cm	48 cm
Birth weight	1,680 g	3,080 g
Birth head circumference	30 cm	33.5 cm
Current height	160 cm	161 cm
Current weight	44.2 kg	47 kg
Current head circumference	50 cm	53 cm
Heart defect^b^	Mild PS	None
Infantile hypercalcemia	No	No
Kidney abnormalities	None	None
Developmental anomalies	Bilateral inguinal hernia and ectopic testis	Bilateral inguinal hernia and cutaneous cysts
Facial dysmorphism	Typical WBS features	Typical WBS features
Walking^c^	Unstable	Unstable
Neurological examination	Unremarkable	Brisk reflexes

^a^*PS* pulmonary stenosis, *WBS* Williams-Beuren syndrome. ^b^For patient 1, a heart murmur was detected at 3 weeks old, and the diagnosis of mild PS was made by cardiologists; for patient 2, a systolic heart murmur was detected at 1 year old, but there was no heart defect. ^c^Patient 1 had an abnormal gait, needed assistance to maintain balance when standing upright and required hand-holding when walking uphill or downhill; patient 2 also had balance disturbances and an abnormal gait (head forward and bent back like his father and brother).

Patient 1, a 17.6-year-old male, was born prematurely at 32 weeks of gestation. The pregnancy was complicated by maternal depression. Patient 1 had feeding difficulties in infancy related to a gastroesophageal reflux. According to parental report, sleep disturbances were present since the first year of life and included longer sleep latency associated with body rocking in the bed before sleeping and very deep sleep. The current presence of these disturbances was confirmed by Actiwatch monitoring (Mini Mitter Co, Bend, OR, USA). The patient first walked at 3.5 years old with balance disturbances, never developed expressive language and was not toilet-trained (no bladder or bowel control). The parents and the pediatrician noticed autistic behaviors during the first months of life, such as absence of eye contact and absence of facial expressions directed toward others. Referral to a child-care center for autistic disorder occurred at 3.5 years of age. On the basis of dysmorphic facial features (large forehead, broad nose and long philtrum, dental malocclusion) and mild pulmonary stenosis, a clinical diagnosis of WBS was initially suggested in infancy, but cytogenetically confirmed only during our systematic genetic survey 15 years later.

Patient 2, a 19.2-year-old male, was born after a full-term pregnancy with no neonatal complications. On the basis of parental report, sleep disturbances were present since infancy and included nocturnal and early morning awakenings (current presence confirmed by Actiwatch monitoring). The patient first walked at 17 months but kept an unsteady gait. He never developed expressive language, and sphincter control was acquired only between ages 12 and 15 years (daytime bladder and bowel control at 12 years, nighttime bladder control at 15 years). Autistic behaviors, such as absence of social smile and vocalization directed toward others, were noticed by the parents during the first year of life and reported by the pediatrician shortly before 3 years of age. The mother kept him at home until he was referred to a child-care center for autistic disorder at the age of 5. As with patient 1, a diagnosis of WBS was made only during our systematic genetic survey involving a clinical genetic examination.

The clinical genetic examination showed growth retardation for both patients (see Table 1 for birth and current height, weight and head circumference). Cardiac examination, including echocardiography, revealed mild pulmonic valve stenosis in patient 1 and was negative for patient 2. Dysmorphic features were observed in patient 1, including dental malocclusion and typical WBS facial dysmorphism (hypertelorism, short nose with anteverted nostrils, thick lips with everted lower lip and sagging cheeks). Patient 2 had also the characteristic facial features of WBS (elfin-like face). For both patients, the neurological examination showed balance disturbances and an abnormal gait (Table 1). In addition, brisk reflexes were observed for patient 2.

### Behavioral and cognitive assessments

Diagnostic and behavioral assessments were performed using the Autism Diagnostic Interview–Revised (ADI–R) and the Autism Diagnostic Observation Schedule (ADOS) scales [26] (Table 2).

**Table 2:** Autistic impairments and neurobehavioral characteristics

**Characteristics**	**Patient 1**	**Patient 2**
Autistic behavioral domains and autism diagnosis^a^		
ADI–R		
Abnormalities of development evident at or before age 36 months (cutoff = 1)	5	4
Nonverbal communication (cutoff = 7)	14	12
Reciprocal social interaction (cutoff = 10)	26	23
Repetitive behaviors and stereotyped patterns (cutoff = 3)	9	9
ADOS (module 1)		
Communication (cutoff = 4)	5	5
Reciprocal social interaction (cutoff = 7)	13	8
Communication + social interaction total (cutoff = 12)	18	13
Play	3	1
Repetitive behaviors and stereotyped patterns	5	4
Expressive behaviors		
Verbal language	None (total absence of words)	None (total absence of words)
Self-injurious behaviors	Ear-slapping, hand-biting	Self-scratching until bleeding, elbow-banging

^a^According to the ADI–R (parental interview) or the ADOS (direct observation of the patient) algorithm, the DSM-IV-TR and the ICD-10 diagnostic criteria for autistic disorder are fulfilled when the total scores of each domain reach the cutoff point.

The ADI–R is an extensive semistructured parental interview, and the ADOS involves direct observation of the patient through a standardized semistructured situation of games. The ADI–R and the ADOS were administered by two trained psychiatrists certified in the administration of these scales. The ADI–R and ADOS scales were used to assess the three major domains of autistic impairment: reciprocal social interactions, verbal and nonverbal communication, stereotyped behaviors and restricted interests. We used ADOS module 1, which is dedicated to individuals with no or limited speech. The severity of impairment in the major domains and subdomains of autism were scored using the subset items included in the ADI–R and ADOS algorithms. The ADI–R and ADOS items are scored on an ordinal scale from 1 to 3 according to autism severity, with 0 coding meaning that the autistic behavior was not present. Interjudge reliability with respect to the critical distinction between mild, moderate and severe impairment was excellent, with an interjudge agreement of 94% between the two expert raters. The ADI–R scale is validated to assess the behavior that was most abnormal during the 4- to 5-year-old period and the current behavior. The ADI–R algorithm is based on the 4- to 5-year-old period of life. We also reported the ratings of the subset of ADI–R items included in the ADI–R algorithm for the current period to see the evolution between these two periods of life (Table 3). In addition, the ADI–R and the ADOS were coded independently, but the ratings reported in Table 3 for the current period took into consideration the actual direct observation of the patient during the ADOS administration when a competency was observed. This approach, combining multiple sources based on the clinician’s judgment and the administration of the ADI–R completed by the ADOS, is recommended [27] and improves the confidence one can have in the current period assessment.

**Table 3:** **Assessment of the autistic behavioral domains and subdomains based on the Autism Diagnostic Interview–Revised algorithm**^**a**^

**Assessment**	**Patient 1**		**Patient 2**	
	**Ages 4 to 5 years**	**Age 17 years**	**Ages 4 to 5 years**	**Age 19 years**
Total social interaction	2	1	2	1
B1: Failure to use nonverbal behaviors to regulate social interaction	2	1	2	1
B2: Failure to develop peer relationships	2	1	3	1
B3: Lack of shared enjoyment	3	2	3	1
B4: Lack of socioemotional reciprocity	2	1	2	1
Total nonverbal communication	3	2	3	1
C1: Delayed spoken language and failure to compensate through gesture	2	2	2	1
C4: Lack of varied spontaneous make-believe or social imitative play	3	1	3	0
Total repetitive behaviors and stereotyped patterns	3	2	2	2
D1: Encompassing preoccupation or circumscribed pattern of interest^b^	3	2	2	2
D2: Compulsive adherence to nonfunctional routines or rituals	1	1	2	0
D3: Stereotyped and repetitive motor mannerisms^c^	3	2	3	3
D4: Preoccupations with part of objects or nonfunctional elements	2	1	1	2

^a^The scoring has been transformed in median values as previously described [45] (0: autistic behavior is not present, 1: mild impairment, 2: moderate impairment, and 3: severe impairment). ^b^Patient 1 showed unusual repetitive sensory interests, such as fascination for spinning objects and water in the shower, sniffing people, actively seeking “wind sensations” (manual or electric fan) and interest in musical objects with hyperresponsivity to auditory stimuli. Patient 2 showed unusual repetitive sensory interests, such as fascination with spinning objects and water in the bath, sniffing people and interest in musical objects with hyperacusis to loud sounds. (He covered his ears at screams, babies crying and sounds of an airplane). ^c^Patient 1 showed finger mannerisms and head-rocking, and patient 2 showed hand-flapping and toe-walking.

Cognitive functioning was assessed by a psychologist using the age-appropriate Wechsler Adult Intelligence Scale III (WAIS-III) [28] and the Kaufman Assessment Battery for Children (K-ABC) [29], given the interest of the K-ABC in nonverbal individuals, especially in language-disordered children.

### Molecular analyses: single-nucleotide polymorphism array and *SLC6A4* genotyping

DNA was isolated from peripheral blood lymphocytes by following standard procedures. We excluded atypical rearrangement by single-nucleotide polymorphism (SNP) array genotyping. For patient 1, a whole-genome SNP array was performed using Illumina HumanCytoSNP-12 v1.0 DNA Analysis BeadChip Kit (Illumina, San Diego, CA, USA) and analyzed using Illumina GenomeStudio software (Genotyping 1.1.9, Genome Viewer 1.1.11) with copy number variation (CNV Partition 2.3.4) according to the manufacturer’s recommendations. The reference used was genome build 36.1. For patient 2, a whole-genome SNP array was performed using Illumina HumanCNV370-Quad v3.0 software and analyzed using IlluminaBeadStudio v2009. The reference used was genome build 36, according to the manufacturer’s recommended protocol. Slightly different whole-genome SNP array procedures were used due to the laboratory’s change to the new protocol.

In addition, genotyping with respect to the promoter polymorphism (serotonin transporter–linked polymorphic region, 5-*HTTLPR*) of the serotonin transporter gene locus *SLC6A4* (solute carrier family 6 (neurotransmitter transporter, serotonin), member 4) was performed using the polymerase chain reaction (PCR) primers 5′-ATGCCAGCACCTAACCCCTAATGT-3′ (position −1,400 to −1,377) and 5′-GGACCGCAAGGTGGGCGGGA-3′ (position −1,001 to −982). These primers gave two products after PCR amplification, a short variant (*s*) of 375 bp and a long variant (*l*) of 419 bp. Analysis followed standard protocol.

### Determination of blood serotonin and urinary 6-sulfatoxymelatonin

Levels of platelet serotonin were determined in whole blood by high-performance liquid chromatography according to a previously described method with a day-to-day coefficient of variation of 5.9% [30]. Secretion of melatonin, a pineal gland hormone produced from serotonin, was assessed by measuring the overnight urinary output of the predominant melatonin metabolite, 6-sulfatoxymelatonin (6-SM). It is well-established that urinary excretion of 6-SM gives an accurate reflection of pineal melatonin secretion [31]. Nocturnal urine was collected at home by caregivers during a 12-h period (from 8 PM to 8 AM). Collected urine samples were stored in a refrigerator until delivered to the laboratory within 24 h of collection. The volume of the urine collection was measured, and a portion was frozen until analyzed for 6-SM. Blinded analysis of urine 6-SM levels was performed by radioimmunoassay using an assay kit from Stockgrand Ltd (Guildford, UK). The urine samples were diluted prior to assay (1:250). The intra-assay coefficient of variation was 6% for a 0.030 μg/ml control sample value. Excretion of 6-SM was expressed as micrograms excreted per hour over the collection period. The urinary 6-SM concentration (μg/ml) was multiplied by the collection volume in milliliters and divided by the 12 hours of collection.

## Results

### Characterization of the deletion by single-nucleotide polymorphism array

The molecular characterization by fluorescence in situ hybridization indicated that the elastin gene was deleted in both patients. The deletion was further characterized by high-density SNP array. The results are presented in Table 4. Both patients had the common short deletion, with recombination occurring between proximal and medial clusters. For patient 1, the deletion spanned 1.41 Mb. For patient 2, the deletion spanned 1.67 Mb. No other additional copy number variation was detected, and no isodisomic segment was identified using a 1-Mb window.

**Table 4:** **Characterization of the Williams-Beuren syndrome critical region deletion by single-nucleotide polymorphism array**^**a**^

**Characteristics**	**Patient 1**	**Patient 2**
Size of the deletion (Mb)	1.41	1.67
Centromeric breakpoint (SNP nt position)		
First hemizygous probe locus	72360917	72229683
Homology block in centromeric LCR	C	B
Effect	Intronic interruption of the *FKBP6* gene	Intronic interruption of the transcription factor IIi, pseudogene 1 (*GTF2IP1*)
Telomeric breakpoint (SNP nt position)		
Last hemizygous probe locus	73772847	73900557
Homology block in medial LCR	B	B
Effect	Intronic interruption of the *GTF2I* gene	Intronic interruption of the *GTF2IRD2* gene

^a^*LCR* low copy repeat, *nt* nucleotide, *SNP* single-nucleotide polymorphism.

### *HTT* allele genotyping

Genotyping with respect to the polymorphism of the promoter of the serotonin transporter gene (*5-HTTLPR*, locus *SLC6A4)* showed that both patients bore the *ss* genotype.

### Behavioral and cognitive phenotypes

The diagnosis of autism based on the experienced clinician’s judgment was confirmed by the ADI–R and ADOS ratings. Indeed, the two patients fulfilled DSM-IV-TR and ICD-10 diagnostic criteria for autistic disorder based on the ADI–R (4- to 5-year-old period) and ADOS (current period) algorithms [26] (see Table 4 for patient information). As shown in Table 3, both patients displayed severe impairment in communication, including a total absence of expressive speech in social interaction (with a positive evolution after the age of 6 years, based on the ADI–R parental interview), and stereotyped behaviors or interests, such as fascination with spinning objects. Autistic behavior was reported for both patients in the first year of life by their parents, and both patients were diagnosed before the age of 3 years with autistic disorder according to Kanner’s criteria [32] and based on a professional’s judgment.

Both patients showed severe intellectual disability. Their verbal IQ scores showed a floor effect (that is, the lowest scores on the norms) related to the total absence of verbal language. Their performance and full-scale IQ scores also showed a floor effect on the WAIS-III scale (verbal IQ = 46, performance IQ = 45, full-scale IQ = 40). On the K-ABC scale, patient 1 had a raw score of 5 on the Triangle subtest (corresponding to the performance of a 4.5-year-old child) but failed the other nonverbal subtests. Patient 2 was able to complete all the nonverbal subtests and obtained a raw score of 10 on the Triangle subtest. On the basis of his performance on the nonverbal scale, patient 2’s performance corresponded to that of a 5-year-old child.

### Serotonin and 6-sulfatoxymelatonin measures

Patients 1 and 2 were both markedly hyperserotonemic with whole-blood serotonin levels of 440 ng/ml and 370 ng/ml, respectively (mean ± SD value in healthy controls: 152 ± 50 ng/ml) [21]. In addition, both patients exhibited substantially reduced nocturnal urinary 6-SM excretion rates (patients 1 and 2: 0.57 μg/h and 0.64 μg/h, respectively) compared to healthy controls (mean value ± SD: 1.80 ± 1.59 μg/h) [22].

## Discussion

Herein we report on two males originally recruited to participate in a genetic study of autism who were discovered, upon clinical and molecular examination, to have classic WBSCR deletions. Although both individuals had the typical WBS facies and exhibited sleep problems often seen in WBS patients, their behavioral phenotype stood in contrast to that typical of people with WBS. Quite unlike the common WBS profile of talkativeness, overfriendliness and mild to moderate intellectual disability, both patients displayed severe impairments in verbal communication and social interaction, together with severe intellectual disability, and, more specifically, met the criteria for autism. In addition, both patients exhibited platelet hyperserotonemia and low nocturnal urinary melatonin excretion, neurochemical phenotypes associated with autism [20,22,23,33]. Thus, behavioral and neurochemical phenotypes typically associated with autism were observed in these two patients with WBS.

The presence of autistic behavioral and neurochemical phenotypes in WBS patients could be due to the co-occurrence in these individuals of the WBSCR deletion with other genetic and/or environmental risk factors for autism, or it could reflect an alternate expression of WBS. Only 13 patients have previously been reported in four countries other than France (six in the United States, four in Sweden, two in Germany and one in Turkey) to have the common WBSCR deletion while also meeting criteria for autistic disorder [34-39], making the first explanation plausible. However, we reported recently on nine French individuals with autistic disorder associated with WBS, suggesting that comorbid autism and WBS is more frequent than expected [40]. Furthermore, some studies [38,41] have reported social communication deficits in individuals with common WBSCR deletions, suggesting that impairment in social communication might be associated with WBS more frequently than expected and tending to favor the alternate expression explanation. Thus, verbal language in individuals with WBS appears not to be socially adapted and relevant to social communication. Our findings, in line with these studies, suggest that abnormal social communication in WBS patients forms a continuum ranging from classic excessive talkativeness and overfriendliness to absence of verbal language and poor social relationships.

Taking into account that both patients had common WBSCR deletions, such a large variation in the behavioral expression of the WBSCR deletion is not easily explained. One explanation could rely on the intrinsic variability in phenotypic expression in the theoretical context of a polygenic, multifactorial model. A second possible model is a trans-acting effect, that is, a mutation or polymorphism in one of the genes present in the retained WBSCR. In this regard, the genes of the *GTF2* family of transcription factors are good candidates for modifiers of the WBS phenotype. Thus, several authors have suggested that *GTF2I* hemizygosity could be a major cause of intellectual disability in patients with WBS [7,42]. It is noteworthy that this gene has been related to language [4]. Alteration in the expression of genes flanking the breakpoints by positional effect, mediated by a *cis*-acting mechanism, is an attractive third hypothesis [43]. Differences in the precise breakpoints could modify expression of nonhemizygous flanking genes. Genes flanking the deletion could have their expression altered by the rearrangement and participate in the phenotype even if they do not vary in copy number [44]. However, molecular characterization of the deletion in our two patients excludes the possibility of an atypical genomic rearrangement.

The phenotypic variability observed in patients with deletion of the WBSCR could also result from interaction with other genetic, environmental or stochastic factors. One possible modifying or epistatic locus is the *5-HTTLPR* allele of the serotonin transporter (*5-HTT*) gene. Both patients were homozygous for the s allele. Although *5-HTTLPR* alleles have not been consistently associated with autism per se, several studies have reported that the *s* allele is associated with the most severe autistic impairment in communication and social interaction [45-47]. Thus, allelic transmission in autism probands was dependent upon the severity of impairments in the social and communication domains, with greater s allele transmission in severely impaired individuals. This relationship between s alleles and the severity of autistic impairment was also seen when ratings of social and communication behaviors were compared across ss, ls and ll genotypes [45]. These findings are consistent with the suggestion that the *ss* genotype generally has deleterious effects on human and nonhuman behavior [48-50]. The observation that infants homozygous for the *s* allele have lower orientation and alertness scores [51] may also be relevant to an influence of the *HTT* gene on the phenotypic spectrum of WBS. In addition, the reported association of the *HTT* promoter polymorphism with anxiety-related traits in the general population [52] further stimulates interest in *HTT* as a possible candidate gene to modify the WBS behavioral phenotype. Given that the frequency of the *s* allele in the general population (in samples of predominately northern European ancestry) is as high as 43% [52,53] (approximately 18% homozygosity), one would expect to find a substantial proportion of WBS patients with the *ss* genotype. The probability that any two WBS patients would both have the *ss* genotype is approximately 0.18 × 0.18, or 0.032. The co-occurrence of WBS and autistic disorder might be underestimated, given the nondetection of autistic social communication impairments in individuals with the typical WBSCR deletion [38,41]. This raises issues related to fundamental difficulties in making the diagnosis of autistic disorder, especially when associated with genetic disorders and intellectual disability [54,55]. In addition, the search for behaviors related to autism spectrum disorders in patients with WBS may have important clinical implications, considering that some children with WBS could benefit from therapies offered to children with autistic disorder. Similarly, the frequency of WBS among children with autistic disorder might be underestimated, considering that, based on our experience in France, practitioners working with children with autistic disorder do not ask routinely for a clinical genetic examination with a clinical morphological examination to search for genetic disorders associated with autism, including WBS. This situation highlights the need to conduct systematically a clinical genetic examination to search for underlying genetic disorders in all individuals with an autistic behavioral phenotype.

The presence of frank hyperserotonemia and lower melatonin production in patients 1 and 2 is intriguing. Previous investigators who studied measurements of platelet serotonin in two WBS patients with hyperserotonemia also reported autistic behavior, including severe deficit in verbal communication with typical autistic language impairments, such as echolalia or repetitive and noncommunicative vocalizations [34]. An absence of such behavior in WBS patients with normal serotonin levels has been reported [56]. The hypothesis of a possible role of hyperserotonemia in autistic behavior associated with WBS is strengthened by the well-replicated high frequency of hyperserotonemia in children with autistic disorder (25% to 50% according to many studies, whereas the frequency of hyperserotonemia in the general population is about 2.3%) [21]. It is noteworthy that hyperserotonemia is a characteristic neurochemical phenotype associated with autism, but is not specific of this disorder (hyperserotonemia has been observed, for example, in non-autistic individuals with intellectual disability since the Schain and Freedman first study in 1961 [57]). Hyperserotonemia might be related less to the nosographic category of autism than to a dimensional impairment, notably in the verbal communication dimension. Thus, several studies have found significant negative associations between hyperserotonemia and verbal expressive abilities in individuals with autistic disorder [58,59]. It can be speculated that hyperserotonemia and/or genetic variation contributing to hyperserotonemia might influence the behavioral expression of the WBS phenotype, especially in the verbal communication domain, with a possible total absence of expressive language persisting even during adulthood. In this regard, the role of the *GTF2IRD1* gene might be of interest considering that mice with disruption of *GTF2IRD1*, used as a behavioral animal model of WBS, showed enhanced prefrontal 5-HT1A-mediated inhibitory responses following bath applications of 5-HT [60]. This finding might be relevant to the disinhibition observed in individuals with WBS and their hyperensitivity to serotonergic medicines such as the selective serotonin reuptake inhibitors (SSRIs) [61]. Given blood–brain barrier considerations, it seems doubtful that peripheral platelet hyperserotonemia could directly affect neurodevelopment. However, given the critical early role of serotonin in embryonic and neuronal development and the presence of apparent serotonergic abnormality (hyperserotonemia) in autistic behavior associated with WBS, a role for serotonin is worth considering. At the least, it can be suggested that platelet serotonin levels might offer a potential predictor of an autism behavioral phenotype in WBS. It might also be of interest that epigenetic mechanisms involving gene X environment interactions and effects on the serotoninergic system [62] have been found in several genetic syndromes associated with autism spectrum disorders [63]. Further studies on gene X environment interactions, including epigenetic mechanisms, are necessary to better understand the association between common WBSCR deletions and typical autistic behavioral and neurochemical phenotypes.

## Conclusions

In two individuals, we found the classical WBSCR deletion to be associated with severe autism, including a total absence of expressive language, which usually is not reported for individuals with common WBS. Future research is required to ascertain the mechanisms underlying this association between WBS and severe autistic disorder and to better understand the influence of genetic background and specific epistatic effects in WBS. The possible role of serotonin-related genes and serotonergic abnormality in the expression of social and communication behaviors in WBS may be of particular interest. Further studies are necessary to document the possible association of hyperserotonemia and/or *HTT* alleles as modifiers of the WBS neurocognitive profile in larger cohorts of individuals with WBS, with or without autistic disorder. From this perspective, WBS provides a challenging but relatively well-defined and potential fruitful model to develop integrated genetic, cognitive, behavioral and neurochemical approaches to study genotype–phenotype correlations as well as possible gene–environment interactions and genetic background effects.

## Abbreviations

ADI–R: Autism Diagnostic Interview–Revised; ADOS: Autism Diagnostic Observation Schedule; CNV: Copy number variation; DSM-IV-TR: Diagnostic and Statistical Manual of Mental Disorders, Fourth Edition*–*Text Revision; ELN: Gene coding for elastin; GTF2I: Gene coding for general transcription factor IIi; GTF2IRD1: General transcription factor IIi repeat domain–containing protein 1; HTT gene: Serotonin transporter gene; 5-HTTLPR: Promoter of the serotonin transporter gene; ICD-10: International Classification of Diseases, 10th Revision (Mental and Behavioral); K-ABC: Kaufman Assessment Battery for Children; 5- LCR: Low copy repeat; SNP: Single-nucleotide polymorphism; 6-SM: 6-sulfatoxymelatonin; SSRI: selective serotonin reuptake inhibitor; WAIS-III: Wechsler Adult Intelligence Scale III; WBS: Williams-Beuren syndrome; WBSCR: Williams-Beuren syndrome critical region.

## Competing interests

The authors declare no competing financial interests.

## Authors’ contributions

ST and AV designed the study. ST, DC, CC and MC recruited the participants. ST and SK performed the behavioral assessments. AV, PSV, CL and YT performed the biological measurements. ST, AV, GMA, DC and MC wrote the article. All authors read and approved the final manuscript.
